# First effectiveness data of lenvatinib and pembrolizumab as first-line therapy in advanced anaplastic thyroid cancer: a retrospective cohort study

**DOI:** 10.1186/s12902-024-01555-y

**Published:** 2024-02-22

**Authors:** Dominik Soll, Philip Bischoff, Anne Frisch, Marie Jensen, Zehra Karadeniz, Martina T. Mogl, David Horst, Tobias Penzkofer, Joachim Spranger, Ulrich Keilholz, Knut Mai

**Affiliations:** 1 Department of Endocrinology and Metabolism, Charité Universitätsmedizin Berlin, Corporate Member of Freie Universität Berlin, Humboldt-Universität Zu Berlin, Luisenstr. 13, 10117 Berlin, Germany; 2 Institute of Pathology, Charité Universitätsmedizin Berlin, Corporate Member of Freie Universität Berlin, Humboldt-Universität Zu Berlin, Berlin, Germany; 3 Berlin Institute of Health at Charité - Universitätsmedizin Berlin, Berlin, Germany; 4 German Cancer Consortium (DKTK) and German Cancer Research Center (DKFZ), Heidelberg, Germany; 5 Department of Radiology (CVK), Charité - Universitätsmedizin Berlin, Corporate Member of Freie Universität Berlin, Humboldt-Universität Zu Berlin, 13353 Berlin, Germany; 6 Department of Endocrinology and Metabolism, Charité - Universitätsmedizin Berlin, Corporate Member of Freie Universität Berlin, Humboldt-Universität Zu Berlin, 10117 Berlin, Germany; 7 Department of Surgery, Campus Charité Mitte | Campus Virchow-Klinikum, Charité - Universitätsmedizin Berlin, Corporate Member of Freie Universität Berlin, Humboldt-Universität Zu Berlin, Berlin, Germany; 8 Charité-Center for Cardiovascular Research (CCR), Berlin, Germany; 9 DZHK (German Centre for Cardiovascular Research), Partner Site Berlin, Berlin, Germany; 10 German Center for Diabetes Research (DZD), 85784 Neuherberg, Germany; 11 NutriAct-Competence Cluster Nutrition Research Berlin-Potsdam, 14558 Nuthetal, Germany; 12 Comprehensive Cancer Center, Charité - Universitätsmedizin Berlin, Corporate Member of Freie Universität Berlin, Humboldt-Universität Zu Berlin, Chariteplatz 1, 10117 Berlin, Germany

**Keywords:** Anaplastic thyroid cancer, Lenvatinib, Kinase inhibitor, Pembrolizumab, Cancer immunotherapy

## Abstract

**Background:**

Anaplastic thyroid cancer (ATC) is a rare and aggressive neoplasm. We still lack effective treatment options, so survival rates remain very low. Here, we aimed to evaluate the activity of the combination of lenvatinib and pembrolizumab as systemic first-line therapy in ATC.

**Methods:**

In a retrospective analysis, we investigated the activity and tolerability of combined lenvatinib (starting dose 14 to 24 mg daily) and pembrolizumab (200 mg every three weeks) as first-line therapy in an institutional cohort of ATC patients.

**Results:**

Five patients with metastatic ATC received lenvatinib and pembrolizumab as systemic first-line therapy. The median progression-free survival was 4.7 (range 0.8–5.9) months, and the median overall survival was 6.3 (range 0.8-not reached) months. At the first follow-up, one patient had partial response, three patients had stable disease, and one patient was formally not evaluable due to interference of assessment by concomitant acute infectious thyroiditis. This patient was then stable for more than one year and was still on therapy at the data cutoff without disease progression. Further analyses revealed deficient DNA mismatch repair, high CD8^+^ lymphocyte infiltration, and low macrophage infiltration in this patient. Of the other patients, two had progressive disease after adverse drug reactions and therapy de-escalation, and two died after the first staging. For all patients, the PD-L1 combined positive score ranged from 12 to 100%.

**Conclusions:**

The combination of lenvatinib and pembrolizumab was effective and moderately tolerated in treatment-naïve ATC patients with occasional long-lasting response. However, we could not confirm the exceptional responses for this combination therapy reported before in pretreated patients.

**Supplementary Information:**

The online version contains supplementary material available at 10.1186/s12902-024-01555-y.

## Introduction

Anaplastic thyroid carcinoma (ATC) is the rarest type of thyroid cancer with an incidence of approximately 1/1.000.000/year [1]. Unlike most other kinds of thyroid cancers, it is an aggressive disease with an extremely poor prognosis and a 5-year-survival of less than 10% due to lack of effective treatment options for advanced disease [1]. At time of diagnosis, nearly 50% of the patients have distant metastases [2]. Until recently, systemic therapy was limited to chemotherapy with very low response rates and short-lasting efficacy [3].

However, targeted therapy approaches have recently shown remarkable results leading to improved survival rates in the respective subgroups of patients [4]. Effective therapies are available for ATC subtypes that possess a *BRAF* V600E mutation or certain gene fusions. Novel therapeutic strategies for ATCs without identified driver alterations are currently under investigation. Iyer et al. showed that the PD-1 inhibitor pembrolizumab can be effective as an additional salvage therapy after disease progression under multikinase inhibitor therapy [5]. Recently, a retrospective analysis demonstrated a significant therapeutic effect of combined treatment with lenvatinib and pembrolizumab in ATC patients after previous chemotherapy. Remarkably, the response rate was high, with four out of six patients experiencing a partial response and exceptionally long response durations with a median progression-free survival (PFS) of 16.8 months [6]. Monotherapy using lenvatinib or checkpoint inhibitors has not demonstrated comparable results, respectively [7, 8].

The preclinical rationale for using the anti-angiogenetic kinase inhibitor lenvatinib with immunotherapy for ATC has recently been reviewed by Boudin *et al *[9]. Lenvatinib targets VEGF, EGFR, and PDGF receptors, among others, and is regularly used in differentiated thyroid cancer refractory to radioiodine treatment [9, 10]. While genetic alterations in the VEGF pathway have been associated with aggressiveness in differentiated thyroid cancer, respective data on ATCs is still scarce [11]. However, anti-angiogenetic kinase inhibitors are believed to help overcome resistance to immunotherapy in a variety of solid tumors [12].

Given the rapid progression of the disease, timely initiation of effective therapy is a key element for its successful treatment. Data from *BRAF* V600E-positive tumors and neoadjuvant treatment with dabrafenib and trametinib illustrated that early initiation of an effective treatment can lead to curative therapy options of otherwise incurable diseases in ATC [13, 14]. Moreover, its manifestation at a vulnerable site make ATC prone to additional morbidity [15]. Therefore, promising therapy options in ATC should be investigated as early as possible during the course of the disease. Here, we present real-world outcome data on the activity of the combination of lenvatinib and pembrolizumab as a systemic first-line therapy regimen in ATC.

## Methods

This retrospective cohort study was approved by the Ethics Committee of Charité-Universitätsmedizin Berlin (EA1/238/22). Informed consent was not necessary according to state legislations (Berliner Landeskrankenhausgesetz (LKG) of September 18, 2011, § 25). Reporting followed the Strengthening the Reporting of Observational studies in Epidemiology (STROBE) statement for cohort studies.

### Study design and population

This retrospective cohort study was conducted at a single university healthcare center and data were collected from patients who started therapy between January 2011 and April 2022. Follow-up data were collected until November 2022. All patients who met the following inclusion criteria were included: diagnosis of ATC and treatment with lenvatinib and pembrolizumab as first-line therapy for unresectable disease. The diagnosis was histopathologically confirmed in all patients. In a secondary analysis, we included patients who had ATC and were treated with any other therapeutic agents as systemic first-line therapy to demonstrate the general courses of therapy in a tertiary healthcare center. We revised patients’ charts and obtained available clinical records. Cases with missing follow-up data are indicated.

For all patients who received lenvatinib and pembrolizumab, radiological assessment was based on extensive computed tomography (CT), magnetic resonance imaging (MRI), and/or positron emission tomography (PET)-CT using [^18^F]﻿ fluorodeoxyglucose (FDG). Response to therapy was assessed retrospectively based on the Response Evaluation Criteria In Solid Tumors (RECIST) 1.1 criteria [16]. PFS was defined as the time from the start of treatment until documented progressive disease or death, overall survival (OS) as the time from the start of treatment until death. Tumor size was determined as the sum of the diameters of the target-lesions. Adverse events were graded according to Common Terminology Criteria for Adverse Events (CTCAE) version 5.0.

### H&E and immunohistochemical staining and image analysis

The tissue samples were formalin-fixed, dehydrated and paraffin-embedded. Consecutive tissue section of 2–3 µm thickness were prepared for H&E and immunohistochemical staining. For H&E staining, the sections were stained for 8 min in acidic haemalum staining solution (Waldeck) and for 2.5 min in eosin staining solution (Sigma-Aldrich) using a Tissue-Tek Prisma Plus slide stainer (Sakura). Immunohistochemical staining was performed on Leica Bond III, Leica Bond Max (both Leica Biosystems) and Ventana BenchMark XT (Ventana) immunostainers according to standard protocols provided by the manufacturer. Tissue sections were subjected to heat-induced antigen retrieval and endogenous peroxidase blocking, and subsequently incubated with primary antibodies for 30 min at room temperature. The following antibodies were used: Rabbit anti-CD3 (polyclonal, Cat. No. A045201-2, 1:100, Dako/Agilent), Mouse anti-CD8 (clone C8/144B, Cat. No. M7103, 1:100, Dako/Agilent), Mouse anti-CD68 (clone PG-M1, Cat. No. M0876, 1:200, Dako/Agilent), Mouse anti-MLH1 (clone M1, Cat. No. 790–5091, ready-to-use dilution, Roche/Ventana), Mouse anti-MSH2 (clone G219-1129, Cat. No. 790–5093, ready-to-use dilution, Roche/Ventana), Mouse anti-MSH6 (clone SP93, Cat. No. 760–5092, ready-to-use dilution, Roche/Ventana), and Rabbit anti-PMS2 (Clone EPR3947, Cat. No. 760–5094, ready-to-use dilution, Roche/Ventana). Next, the sections were incubated with HRP-conjugated secondary antibody for 30 min at room temperature, followed by incubation with DAB for 8 min and counterstaining with hematoxylin and bluing reagent for 12 min.

For image analysis, the stained tissue sections were digitized using a Pannoramic SCAN 150 scanner (3DHISTECH). Images were analyzed using the QuPath software (version 0.3.2). For the detection of CD3^+^/CD8^+^ cells, the following parameters were used: Detection Image: Optical density sum, Minimum Area: 20 µm^2^, Nucleus DAB OD mean: 0.6, otherwise default settings. The following parameters were used for the detection of CD68^+^ cells: Minimum Area: 20 µm^2^, Cytoplasm DAB OD mean: 0.2, otherwise default settings. The tumor area was annotated by a pathologist. The proportion of positive cells within the tumor area was then calculated.

### Molecular pathological analysis

For panel sequencing, FFPE tumor tissue samples were retrieved and tumor-enriched areas were macrodissected. DNA was extracted using the Maxwell RSC DNA FFPE Kit (Promega, Cat. No. AS1450) and analyzed using the Oncomine Focus DNA Assay (Thermo Fisher Scientific, Cat. No. A35955). The DNA library was generated using the Ion Chef System (Thermo Fisher Scientific, Ser. No. GSS5PR-0102), and sequencing was performed by an Ion Gene Studio S5 Prime (Thermo Fisher Scientific, Ser. No. CHEF00675). Analysis of the data was assessed by Sequence Pilot (JSI medical system, Version 5.4.0). In patient 3, comprehensive genetic testing was performed by an external provider using the QIAseq Targeted Panel during the patient’s treatment.

### Statistical analysis

Descriptive statistics were performed, and graphs created using GraphPad Prism version 5.04 (GraphPad Software). The median and range were given for continuous variables, and frequency and percentage were given for categorical variables.

## Results

Between January 2011 and April 2022, 13 patients with advanced unresectable ATC were treated with systemic therapy at our institution. Of these, five patients have received lenvatinib and pembrolizumab as systemic first-line treatment (Table 1) between October 2020 and November 2022. Lenvatinib was started at 24 mg daily in three patients and at 14 mg daily in two patients. Pembrolizumab was administered at a dose of 200 mg every three weeks. All five patients received previous or concomitant external beam radiation therapy (EBRT). Patient 1 received palliative EBRT for symptomatic bone lesions with 30–33 Gy per lesion. Patients 2–5 received palliative EBRT for cervical lesions in order to accomplish rapid local tumor control. Here, EBRT was started shortly before or after initiation of systemic therapy (after a median of 14.5 (range -4–32) days) with a median of 41.5 (range 39–45) Gy. None of the patients underwent previous chemotherapy or curatively intended surgery, but all patients underwent diagnostic surgery. Follow-up examinations were available for all patients, and no patient was lost to follow-up at the data cutoff. Most patients had ATC stage IVC with lymphatic or pulmonary metastases (Table 1).

**Table 1:** Baseline characteristics of patients with anaplastic thyroid cancer who received lenvatinib and pembrolizumab as first-line therapy

	Pembrolizumab + Lenvatinib (*n* = 5)
**Age, median (range), years**	65 (52–72)
**Sex, No. (%)**	5
w	2 (40)
**Year of diagnosis, median (range)**	2021 (2020–2021)
**ECOG performance status, No. (%)**	
0	3 (60)
1	1 (20)
2	0 (0)
3	1 (20)
**UICC stage at time of diagnosis, No. (%)**	5
IVA	0 (0)
IVB	1 (20), Patient 3
IVC	4 (80)
**Hypothyroidism with levothyroxine substitution,** **No. (%)**	3 (60)
**Locations of metastases at time of diagnosis, No. (%)**	5
Lymph nodes	4 (80)
Pulmonary	4 (80)
Hepatic	1 (20)
Bone	3 (60)
Intestinal	1 (20)
**Previous surgery (as per intention), No. (%)**	5
diagnostic	4 (80)
curative	0 (0)
other	1 (20), Patient 3: lobectomy + abscess drainage
**EBRT, No. (%)**	5 (100)

*EBRT* external beam radiotherapy, *UICC* Union internationale contre le cancer

**Fig. 1:**
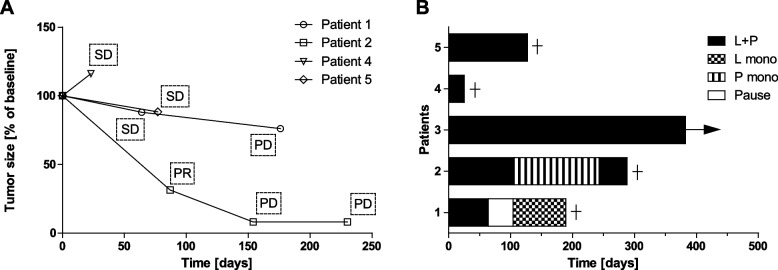
Development of tumor mass and course of therapy of patients with anaplastic thyroid cancer who received lenvatinib and pembrolizumab as first-line therapy. **A** Spider-plot of change in total tumor mass after initiation of combination therapy with lenvatinib and pembrolizumab. Tumor size was determined by the sum of diameters of target-lesions. **B** Time on therapies for patients initially receiving lenvatinib and pembrolizumab. L + P: lenvatinib and pembrolizumab, L mono: lenvatinib monotherapy, P mono: pembrolizumab monotherapy

The median time to the first follow-up radiological assessment was 2.1 (range 0.8–2.9) months. In the first staging, one patient (patient 2) had partial response (PR), three patients (patients 1, 4, 5) had stable disease (SD) and the response in one patient (patient 3) was formally not evaluable as the primary lesion could not be assessed after surgery. For patients 1 + 2, therapy was later de-escalated due to adverse drug reactions. Both patients then experienced disease progression with new lesions, while the size of the baseline lesions continued to decrease (Fig. 1A). Patients 4 + 5 died after the first staging; due to deterioration of the general condition, patient 4 had an early follow-up examination which formally demonstrated stable disease but an increase in tumor size (Fig. 1A). The patient died soon afterwards. Patient 3 initially had concomitant acute infectious thyroiditis and abscess with lobectomy. Formal assessment of the therapy response was hindered by inflammation and surgery; therefore, the response was not evaluable based on RECIST 1.1. An early first radiological follow-up 1.1 months after the initiation of therapy revealed suspect enlarged cervical lymph nodes (LN) without fulfilling the criteria for PD. Therapy with lenvatinib and pembrolizumab was continued, and the lesions responded to treatment or remained stable in the subsequent staging examinations so that the patient continued to be on therapy at the time of data cutoff after more than one year of follow-up without signs of progression (Table 2, Fig. 1B). Overall, the median PFS was 4.7 (range 0.8–5.9) months, and the median OS was 6.3 (range 0.8-not reached) months.

**Table 2:** Response and potential biomarkers of patients with anaplastic thyroid cancer who received lenvatinib and pembrolizumab as first-line therapy

	**Patient 1**	**Patient 2**	**Patient 3**	**Patient 4**	**Patient 5**
**Best overall response**	SD	PR	not evaluable	SD	SD
**Time on therapy, months**	2.1	3.5	ongoing (12.7 +)	0.8	4.2
**Progression-free survival, months**	5.9	5.1	not evaluable	0.8	4.2
**Overall survival, months**	6.3	9.6	ongoing (12.7 +)	0.8	4.2
**Histopathology**					
Ki-67, %	80	26	60	70	30
PD-L1, %					
PD-L1 TPS	15	90	90	10	90
PD-L1 IC	5	0	20	2	< 1
PD-L1 CPS	20	90	100	12	90
DNA mismatch repair status	pMMR	pMMR	dMMR (MLH1- PMS2-)*	pMMR	pMMR
TILs (CD3 +), %	4.4	31.4	14.2	30.0	4.0
CD8 + of TILs, %	72.1	33.8	95.7	60.7	42.9
TAMs (CD68 +), %	40.2	35.2	21.3	37.4	31.8
**Molecular pathology, % allele frequency**	OFA	OFA	QIAseq Targeted Panel	OFA	OFA
*BRAF* p.V600E	-	6	-	-	10
*KRAS* p.G12R	-	-	53	-	-
*TP53* p.P153fs	-	-	56	-	-
*PIK3CA* p.E545K	-	-	-	-	7
**Lenvatinib starting dosage, mg**	14	24	24	24	14

*CPS* combined positive score, *dMMR* deficient DNA mismatch repair, *IC* immune cells, *MSS* microsatellite stable, *OFA* Oncomine Focus Assay, *PD-L1* Programmed cell death 1 ligand 1, *pMMR* proficient DNA mismatch repair, *PR* partial response, *SD* stable disease, *TAM* tumor-associated macrophages, *TIL* tumor-infiltrating lymphocytes, *TPS* tumor proportion score

^*^Molecular pathology of MSS

Adverse events collected from the patients’ clinical records included hypertension (1/5; grade 2), fatigue (2/5; all grade 2), anorexia (3/5; 2 with grade 2, 1 with grade 3), hand-foot-syndrome (1/5; grade 1), diarrhea (1/5; grade 1), proteinuria (4/5; all grade 1), abdominal pain (1/5; grade 1), hemorrhages (1/5; grade 3), tubulointerstitial nephritis (1/5; grade 3), tracheoesophageal fistula (1/5; grade 3), autoimmune hepatitis (1/5; grade 3), and pleural and pericardial effusion (1/5; grade 1). Hypothyroidism newly developed in both patients who were euthyroid before initiation of systemic therapy. Levothyroxine treatment was adapted to achieve euthyroidism. In two patients (patients 2 + 3) who started lenvatinib at 24 mg daily, the dosage was reduced to 14 mg due to anorexia and fatigue. Adverse events that led to further therapeutic adjustments were as follows: a) medication discontinuation after 2.1 months and change to lenvatinib monotherapy after 3.5 months due to drug-induced tubulointerstitial nephritis in patient 1 and b) temporary medication change to pembrolizumab monotherapy after 3.5 months due to tracheoesophageal fistula after a failed attempt to place a percutaneous endoscopic gastrostomy (PEG) tube in patient 2 (Fig. 1B). Later, patient 1 developed new cerebral and intestinal manifestations, whereas patient 2 developed new LN and pulmonary metastases.

We collected additional data from the clinical records and performed immunohistochemical analyses on the patients’ tumor samples to identify potential biomarkers predicting treatment response (Table 2). PD-L1 expression based on the combined positive score (CPS) ranged from 12 to 100% (Table 2). In molecular pathological analyses, two of the five patients (patients 2, 5) demonstrated *BRAF* V600E mutations (Table 2). Interestingly, patient 3 with ongoing treatment had immunohistochemically confirmed DNA mismatch repair deficiency (dMMR) (Fig. S1A), without shifts of microsatellite fragments in capillary electrophoresis. In addition, the proportion of CD8^+^ lymphocytes in all tumor-infiltrating CD3^+^ lymphocytes (TILs) was the highest in this patient, while the proportion of tumor-associated CD68^+^ macrophages (TAMs) was the lowest (Fig. S1B, Table 2). Generally, an association of systemic treatment response with PD-L1 expression could not be derived from our data (Fig. S2).

Finally, we also gathered data of ATC patients who received other systemic therapeutic agents as first-line therapy between January 2011 and 2021. Eight patients were included in this secondary analysis. All patients received cytotoxic chemotherapy regimens (Table S1). Of these, two patients were lost to follow-up, one patient died before the first follow-up, and five patients had available written follow-up data. All these patients had progressive disease in the first follow-up staging after a median time of 2.0 (range 1.6–3.0) months.

## Discussion

In this single-center cohort analysis, we retrospectively assessed the activity of a combined treatment regimen using lenvatinib and pembrolizumab as first-line therapy in patients with ATC. This regimen was a clinically effective systemic first-line therapy in four out of five patients. One patient who received lenvatinib and pembrolizumab was still on therapy after more than 1 year without signs of progression. Two patients experienced disease progression after therapy de-escalation owing to adverse events. Hence, we conclude that response to lenvatinib and pembrolizumab can be long-lasting when the therapy is well tolerated.

To the best of our knowledge, this is the first report on outcome data of lenvatinib and pembrolizumab in treatment-naïve ATC patients demonstrating its activity in combination with local radiation. This expands previous data showing that the combination therapy can be effective in pretreated ATC patients [5, 6]. However, while the first response to therapy was promising in most patients, all but one patient suffered from disease progression within six months. The median OS of 6.3 months found in treatment-naïve patients was comparable with that of Iyer et al*.* who found a median OS of 10.4 months in patients who received pembrolizumab as salvage therapy after progression on lenvatinib [5]. However, it is much shorter than that described by Dierks et al*.* in pretreated ATC patients with a median OS of 16.5 months [6]. A reliable comparison is hindered by the generally low patient count in the studies, which may overly emphasize outliers. Nevertheless, the studies consistently included patients with advanced UICC stage tumors. However, it remains unclear whether early initiation of lenvatinib and pembrolizumab before other systemic therapies is favorable. In melanoma patients, a randomized phase II study recently investigated the benefits of sequencing ipilimumab before or after cytotoxic chemotherapy and found an advantage for immunotherapy before chemotherapy, although the study did not reach full accrual [17]. Based on the presented results, a prospective study to further evaluate the efficacy as a systemic first-line treatment option is warranted. Two prospective trials are ongoing (DRKS00013336, NCT04171622) [18, 19], and we hope to obtain further evidence on the efficacy of lenvatinib and pembrolizumab in treatment-naïve patients and possibly improve patient selection. Additionally, these studies may explore the potential role of the combination therapy in the neoadjuvant setting as it has recently been suggested in two ATC cases [20].To date, there are no validated biomarkers predicting the effectiveness of lenvatinib and pembrolizumab in ATC. However, Dierks et al. found a PD-L1 TPS > 50% or high tumor mutational burden in patients with long-lasting responses [6]. We observed varying PD-L1 expression levels in the presented ATC cases. The immune profile in thyroid cancer samples has recently been extensively phenotyped [21]. Here, ATCs had the highest number of TAMs and cytotoxic CD8^+^ TILs. Similar findings were observed in the present cohort. There has been increasing evidence that TAM infiltration can attenuate the effect of checkpoint inhibitors [22], and a correlation between CD8^+^ TIL infiltration and treatment outcomes was recently demonstrated in a large meta-analysis [23]. Even though the presented data did not allow comparative analysis of long-term and non- or short-term responders, it can be noted that patient 3 with ongoing therapy had the lowest share of TAMs and the highest proportion of CD8^+^ cells within the TILs, in addition to dMMR status. Interestingly, significant and especially durable efficacy of pembrolizumab has recently been shown in MSI-H/dMMR solid tumors regardless of cancer type [24]. Nevertheless, a larger cohort is necessary to dissect the roles of these potential biomarkers for therapy response in ATC.

Interestingly, patients 2 and 5 had *BRAF* V600E mutations. As the *BRAF* state was unclear at time of diagnosis, a treatment regimen including lenvatinib and pembrolizumab was initiated in both patients which has been demonstrated to be effective in a case series of pretreated ATC patients [6] and does not require results from molecular phenotyping. PD-L1 expression was measured but did not influence decision making because its role in predicting efficacy of immunotherapy in ATC remains unclear. Concomitantly, molecular pathology was performed. Our institutional standard procedure comprises an early staging if a *BRAF*-V600E mutation is found. In case of disease progression a switch to BRAF-directed therapy is recommended. However, BRAF-directed therapy for ATC is off-label in Germany. Owing to national legislation, approval and initiation of targeted off-label therapy can be considerably protracted. In case of stable disease or response we prefer to continue lenvatinib and pembrolizumab until disease progression. Patient 2 had PR in the first staging after the initiation of lenvatinib and pembrolizumab. In a later staging where PD was determined in this study (day 154), the interpretation had been different at the time of treatment (ongoing response with “reactive lymph nodes”). Hence, pembrolizumab monotherapy had been continued. When PD was apparent due to new pulmonary lesions (day 230), lenvatinib was reinitiated as bridging therapy and the patient died soon after, before approval for BRAF-directed therapy was granted by health insurance (Fig. 1B). After tumor progression, repeated molecular pathology revealed the *BRAF* V600E mutation in an almost unaltered frequency (from 6% to 2.5%), so that there was no indication of preferential growth of the *BRAF*-mutated clone. In patient 5, BRAF-directed therapy was not initiated since the patient had stable disease in the first staging. In accordance with positive results from a phase 2 trial, the combination of dabrafenib and trametinib is recommended in *BRAF* V600E-positive ATC patients if immediately available at the time point of choosing a therapy regimen [25]. Whether the combination of lenvatinib and pembrolizumab is a valid treatment option in patients with *BRAF*-mutated ATCs remains unclear: while we found transient activity in both patients, a lower objective response rate to the PD-1 inhibitor spartalizumab has previously been demonstrated in ATC patients with *BRAF* V600E mutation [7].

In a secondary analysis of eight historical cases, we found that cytotoxic chemotherapy failed to stop tumor progression in all cases. Nevertheless, the two presented cohorts are too small to draw a reliable comparison regarding treatment efficacy.

The combination of lenvatinib and pembrolizumab can lead to serious adverse events. One patient in this study had tracheoesophageal fistula. In a recent study investigating risk factors for the development of fistulas in patients with lenvatinib for radioiodine-refractory thyroid cancer, EBRT was not found to be significantly associated with the prevalence of fistulas [26]. However, there was a noticeable numerical difference between patients with a fistula (57% with previous EBRT) and those without (36% with previous EBRT). As local radiotherapy in ATC is regularly administered at the time of diagnosis, using lenvatinib and pembrolizumab as systemic first-line therapy might potentially come with a higher risk to develop a fistula than using it later. Still, in a large retrospective analysis EBRT has recently been associated with improved disease-specific survival in ATC [27]. In ATC, local tumor aggressiveness drives complications and mortality. About 20–30% of ATC patients demonstrate airway problems already at initial presentation so that tracheostomy is a common therapeutic consequence to prevent airway collapse and suffocation [28]. Tracheostomy drastically reduces the patients’ quality of life. Despite the potentially higher risk for local adverse effects, lenvatinib and EBRT were combined in the treatment of patients 2–5 with pronounced local tumor extension to achieve rapid local tumor control and to avoid local ATC-mediated complications. However, it is important to point out that patients in this study received doses up to 45 Gy while recent data suggests higher effectiveness of doses above 60 Gy per lesion [29]. This decision was made to reduce the risk for local adverse effects during treatment with the combination of lenvatinib and EBRT. The consequence of calculating the risks and benefits of concomitant ERBT and lenvatinib therapy in ATC still needs to be determined.

Interestingly, proteinuria developed in 4 out of 5 patients while hypertension occurred only in 1 out of 5 patients even though hypertension is often thought to bring about proteinuria. However, lenvatinib inhibits signaling via VEGF receptors 1–3. VEGF inhibition has been shown to induce thrombotic microangiopathy in the kidney [30]. While hypertension can be the principal driver of kidney dysfunction and proteinuria, these side effects have also been described to occur independently in patients treated with tyrosine kinase inhibitors [31].

The presented analysis has obvious limitations due to its retrospective nature and small cohort. In particular, the assessment of adverse events and their grades cannot be as systematic as that in a prospective trial. The low patient count did not allow for further statistical assessment of the therapy response or biomarker relevance. It has to be noted that a median OS of approximately six months with occasional long-term responders is not an uncommon finding in case series on ATC [32, 33]. Nevertheless, we regard the presented findings relevant for two reasons: first, despite promising results in the here presented cohort of treatment-naïve ATC patients who received lenvatinib and pembrolizumab, we were not able to replicate the exceptionally positive data from a case series on heavily pretreated ATC patients [6]. Second, comprehensive characterization of long-term responders may guide researchers and support patient selection in the future.

In summary, we were able to provide first evidence that lenvatinib and pembrolizumab are active in treatment-naïve ATC patients. If the combination is well tolerated, patients may respond long-lastingly. Biomarkers known from other tumor types, such as MMR status, TAM, and CD8^+^ TIL infiltration may play a relevant role in the identification of suitable candidates. We highly endorse performing a prospective randomized controlled trial to investigate the effectiveness of this combination therapy as first-line therapy in ATC, with a focus on the identification of predictive biomarkers.

### Supplementary Information

**Supplementary material 1.**

**Supplementary material 2.**

## Data Availability

The datasets generated and/or analyzed during the current study are available from the corresponding author on reasonable request.
