# Supplementary material for: First effectiveness data of lenvatinib and pembrolizumab as first-line therapy in advanced anaplastic thyroid cancer: a retrospective cohort study
Source: BMC Endocr Disord. 2024 Feb 22;24:25. doi: 10.1186/s12902-024-01555-y (PMC10882904; doi:10.1186/s12902-024-01555-y)
Supplement: Supplementary file 1 — Supplementary material 1. [file 12902_2024_1555_MOESM1_ESM.pptx]

## Slide 1
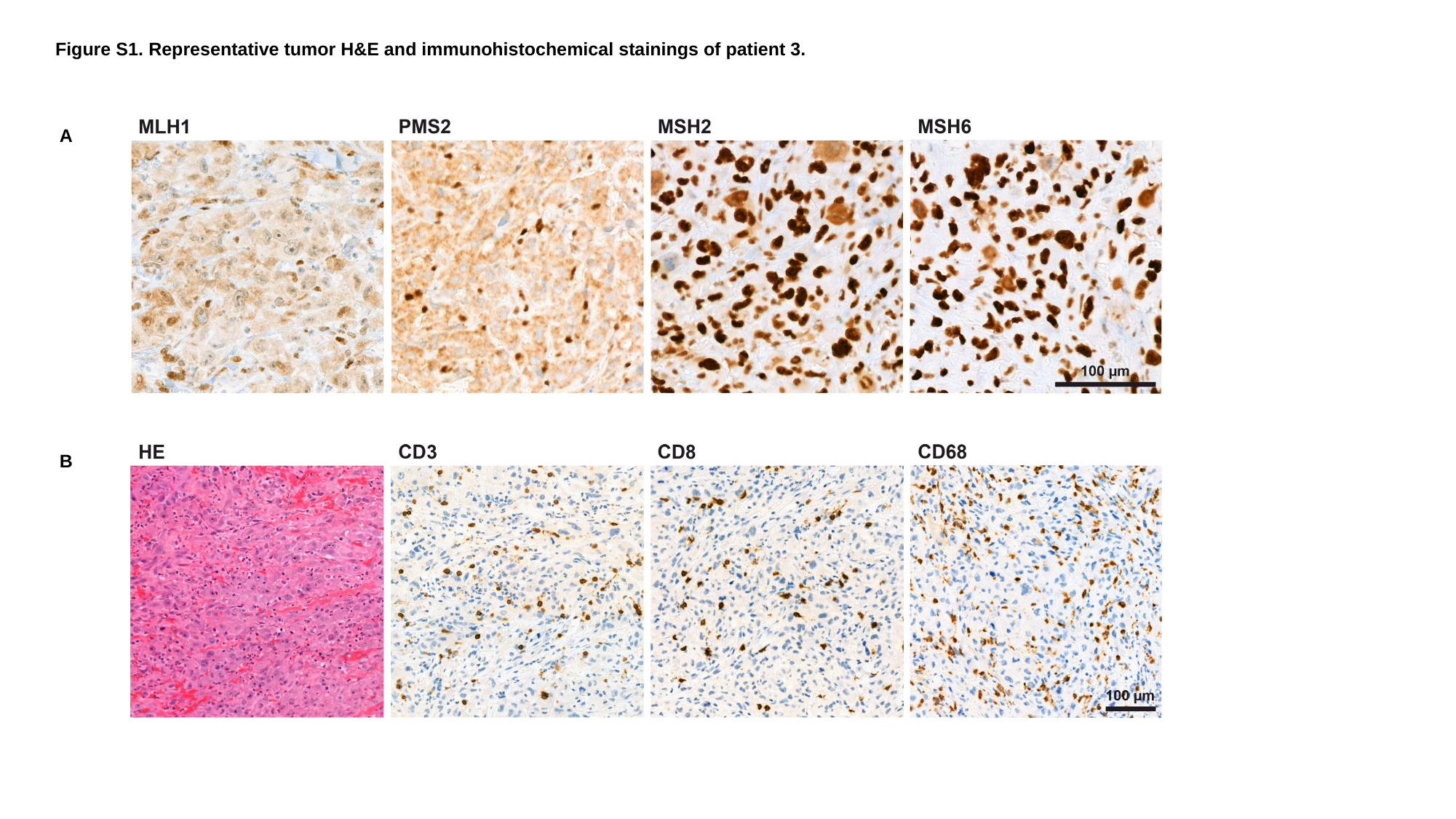

Figure S1. Representative tumor H&E and immunohistochemical stainings of patient 3.
A
B

## Slide 2
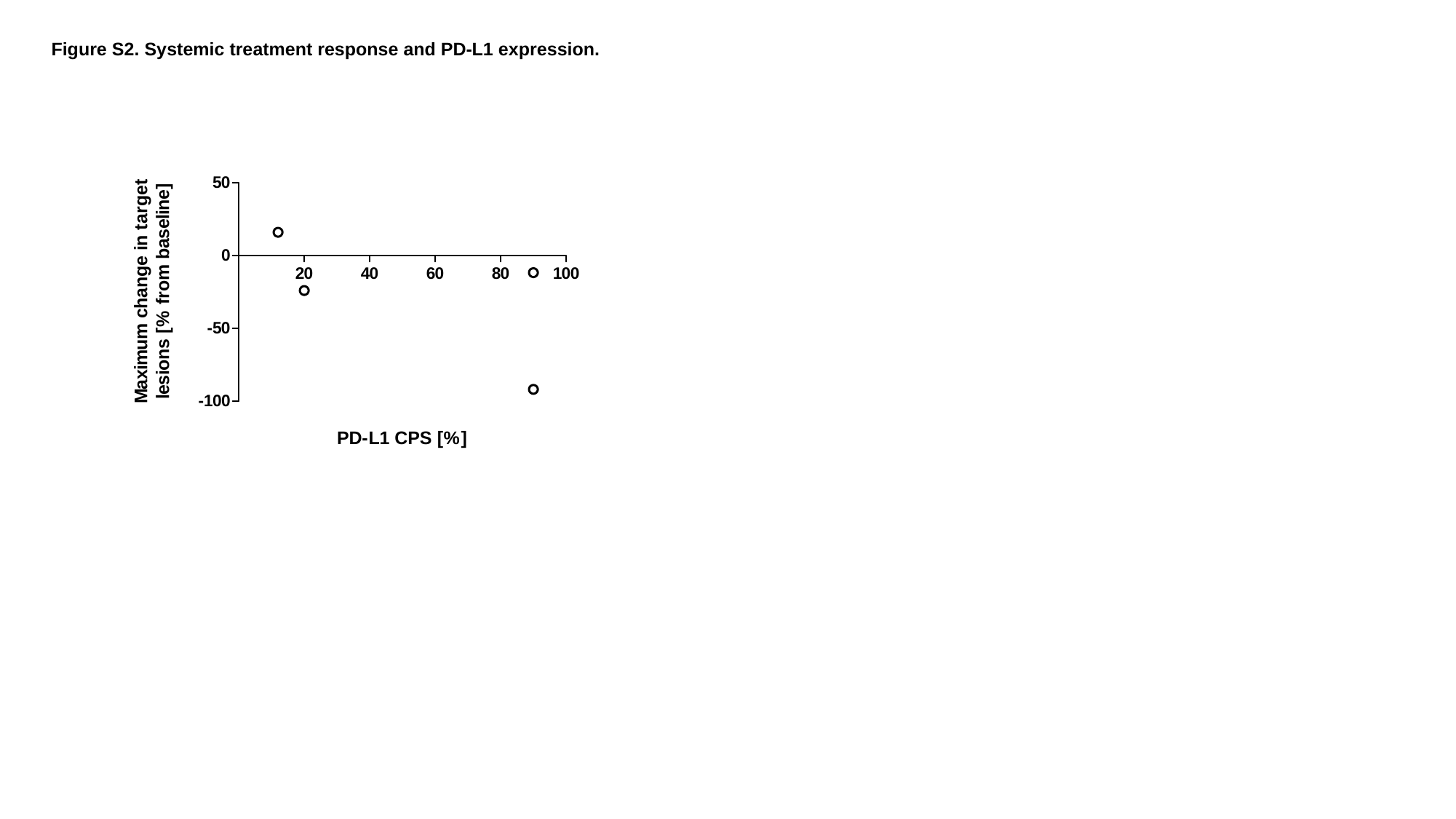

Figure S2. Systemic treatment response and PD-L1 expression.
