# Supplementary material for: First effectiveness data of lenvatinib and pembrolizumab as first-line therapy in advanced anaplastic thyroid cancer: a retrospective cohort study
Source: BMC Endocr Disord. 2024 Feb 22;24:25. doi: 10.1186/s12902-024-01555-y (PMC10882904; doi:10.1186/s12902-024-01555-y)
Supplement: Supplementary file 2 — Supplementary material 2. [file 12902_2024_1555_MOESM2_ESM.docx]

**Supplemental Material**

**First Effectiveness Data of** **Lenvatinib and Pembrolizumab as First-Line Therapy in Advanced Anaplastic Thyroid Cancer: a Retrospective Cohort Study**

**Legends for Figures**

**Figure S1. Representative tumor H&E and immunohistochemical stainings of patient 3.**

Consecutive tissue sections with hematoxylin and eosin (H&E) and immunohistochemical stainings of patient 3. A) Nuclear MLH1 and PMS2 expression is lost in tumor cells while tumor-infiltrating immune cells with retained nuclear expression serve as positive control. Nuclear MSH2 and MSH6 expression is retained in both, tumor and immune cells. B) CD3 and CD8 show the same amount of positive cells.

**Figure S2.** **Systemic treatment response and PD-L1 expression.**

Presentation of radiological response, i.e. maximum tumor shrinkage, and PD-L1 expression in tumor tissue for patients 1, 2, 4, and 5. Formal assessment of the therapy response was not possible for patient 3.

**Table S1. Baseline characteristics of patients with anaplastic thyroid cancer who received cytotoxic chemotherapy as first-line therapy.**

|  | **Chemotherapy (n=8)** |
| --- | --- |
| **Age, median (range), years** | 64 (51-76) |
| **Sex, No. (%)** | 8 |
| w | 3 (37.5) |
| **Year of diagnosis, median (range)** | 2017 (2011-2021) |
| **UICC stage at time of diagnosis, No. (%)** | 8 |
| IVA | 1 (12.5) |
| IVB | 2 (25) |
| IVC | 5 (62.5) |
| **Locations of metastases at time of diagnosis, No. (%)** | 8 |
| Lymph nodes | 5 (62.5) |
| Pulmonary | 5 (62.5) |
| Hepatic | 0 (0) |
| Bone | 0 (0) |
| **Previous surgery (as per intention), No. (%)** | 8 |
| diagnostic | 2 (25) |
| curative | 3 (37.5) |
| other or unknown | 3 (37.5) |
| **EBRT, No. (%)** | 8 (100) |
| **Chemotherapy, No (%)** |  |
| Paclitaxel + Carboplatin | 3 (37.5) |
| Paclitaxel | 1 (12.5) |
| Doxorubicin | 2 (25) |
| Doxorubicin + Docetaxel | 1 (12.5) |
| Doxorubicin + Cisplatin | 1 (12.5) |

EBRT: external beam radiotherapy, UICC: Union internationale contre le cancer.
